# Monogonot rotifers species of the island Cozumel, Quintana Roo, México

**DOI:** 10.3897/BDJ.7.e34719

**Published:** 2019-06-13

**Authors:** Jovana Lizeth Arroyo-Castro, Jesús Alvarado-Flores, Juan Carlos Uh-Moo, Coral Grisel Koh-Pasos

**Affiliations:** 1 Centro de Investigación Científica de Yucatán A.C., Benito Juárez, Cancún, Mexico Centro de Investigación Científica de Yucatán A.C. Benito Juárez, Cancún Mexico; 2 Manejo de Recursos Naturales Isla Cozumel A.C., Cozumel, Mexico Manejo de Recursos Naturales Isla Cozumel A.C. Cozumel Mexico; 3 Ecología de Carreteras, Cozumel, Quintana Roo, Mexico Ecología de Carreteras Cozumel, Quintana Roo Mexico

**Keywords:** Caribbean Sea, zooplankton, island systems, diversity

## Abstract

The current study adds 23 new records to the previously described monogonot rotifers of Cozumel, increasing the number of reported species to 40; these species are grouped into two orders, 11 families and 18 genera. Littoral and limnetic samples from 17 aquatic systems were examined, including wet dolines, coastal lagoons, temporary pools and artificial systems (reservoirs). Of the 36 species found, the following organisms represent new findings for Cozumel: *Anuraeopsis fissa*, *Brachionus angularis*, *B. rubens*, *B. plicatilis* *sensu lato*, *Beuchampiella eudactylota*, *Euchlanis dilatata*, *Mytilina bisulcata*, *Colurella adriatica*, *Lepadella ovalis*, *L. rhomboides*, *Squatinella mutica*, *Lecane aculeata*, *L. climacois*, *L. crepida*, *L. cornuta*, *L. grandis*, *L. obtusa*, *L. pyriformis*, *Cephalodella forficula*, *Scaridium bostjani*, *Trichocerca pusilla*, *Polyarthra cf. dolichoptera*, *P. vulgaris*, *Dicranophorus epicharis* and *Testudinella patina*. Additional information is reported for these species.

## Introduction

Islands contain ecosystems of critical importance for biodiversity conservation since a large number of endemic species are distributed in these environments. Furthermore, islands are important sites for the reproductive, nesting, resting and feeding activities of numerous species, including migratory species (Salazar-Vallejo and González 1993). Despite their great importance, current knowledge about the biodiversity of Mexican islands is limited (Lara-Lara et al. 2008). A Mexican Institute CONABIO-CONANP-TNC-PRONATURA (2007) mentioned that Mexico´s island diversity has not been studied as extensively as terrestrial diversity.

In these ways, several studies on the different taxonomical groups, associated with 149 Mexican islands, have been carried out, in which 2,545 marine species and 2,066 terrestrial species, grouped into 1,830 genera and 655 families, have been reported (CONABIO-CONANP-TNC-PRONATURA 2007). The groups that are studied mostly include birds, algae, fish, reptiles and angiosperms. However, microorganism groups, such as zooplankton and, in particular, rotifers, are not included in these lists even though efforts to understand and increase knowledge about rotifers have intensified in the past two decades. For instance, one inventory of approximately 300 specific rotifer forms (mainly found in fresh water) was created by Elías-Gutiérrez and García-Morales (2011).

Studies have been elaborated mainly for Mexico’s central region, including inventories by Rico-Martínez and Silva-Briano (1993), Sarma et al. (1996), Serranía-Soto (1996), Sarma and Elías-Gutiérrez (1997), Sarma and Elías-Gutiérrez (1998), Sarma and Elías-Gutiérrez (1999) and its south-eastern region (Chiapas, Tabasco, Yucatan and Quintana Roo) in the works of Sarma et al. (2000), Schmitter-Soto et al. (2002), García-Morales and Elías-Gutiérrez (2004), Cervantes-Martínez (2005), García-Morales and Elías-Gutiérrez (2007), Benítez-Díaz et al. (2014). Until now, these studies have focused on continental aquatic systems, leaving behind the advancement of knowledge of this group in island systems. In Mexico, the total area of island systems is 5,083 km^2^ (including small islands, islets, keys and rocks). Cozumel is the third largest island in Mexico (477 km^2^), following the islands of Tiburón and Ángel de la Guarda (Aguirre-Muñoz et al. 2010). In Cozumel, a total of 533 species have been recorded and are distributed between two main groups: aquatic and terrestrial organisms. The aquatic species comprise 68 marine algae, seven freshwater invertebrates, 38 corals, 30 sponges, 102 marine fish and eight freshwater fish. The terrestrial species comprise 40 higher plants, five amphibians, 28 reptiles, 166 birds (resident, endemic and migratory) and 18 mammals (DOF 2012). However, only one taxonomic list of rotifers, cladocera and copepods has been reported for Cozumel by Cervantes-Martínez et al. (2012), who reported 17 rotifer species from 12 sampling sites. For this reason, the present study adds to this list, by presenting an inventory of species richness and new records for the monogonot rotifers.

## Materials and Methods

In Cozumel, 51 biological samples from 17 freshwater systems were analysed. The aquatic system were cenotes, temporal and artificial ponds and mangrove ecosystems. Dissolve oxygen (mg/l), temperature (°C) and electric conductivity (μS/cm), by means of a previously calibrated multi-parametric probe YSI Model 85, were measured in situ and for Max. Depth (m), we used a Secchi disc. Sampling was performed from 2014 to 2016, using a Wisconsin Plankton Net with a 45 µm mesh. The collected material was preserved in alcohol and formaldehyde and live samples were also analysed in the laboratory. The organisms were identified by consulting specialised literature: Koste (1978), Nogrady et al. (1993), Shiel (1995), De Smet and Pourriot (1997), Sarma and Elías-Gutiérrez (1999), Segers (2007), Jersabek and Bolortsetseg (2010), Bertani et al. (2011). In some cases, it was necessary to isolate the trophi dissolving tissues using NaOCl and, after isolation, trophi were washed using distilled water; finally we preserved the trophi(?) semi-permanently (Sarma and Elías-Gutiérrez 1997, Serranía-Soto 1996). Afterwards, the organisms were preserved permanently or semi-permanently, following techniques suggested by Nogrady et al. (1993). The organisms were photographed and illustrated using a camera connected to a high-resolution Zeiss Axio Imager A2 microscope and the AxioVision software SE64 Rel. 4.8. The new records for the state of Quintana Roo, Mexico were deposited in the Reference Collection of The College of the Southern Frontier (El Colegio de la Frontera Sur) under the prefix ECO-CH-Z0. The other taxa were deposited by the authors in a zooplankton collection assigned to the CONACyT project number 2944.

## Results

Four organisms were recorded in Cozumel and the state of Quintana Roo for the first time: *Beauchampiella eudactylota*, *Mytilina bisulcata*, *Squatinella mutica* and *Dicranophorus epicharis*. Overall, a total of 36 monogonot rotifers species were found (see Table 1), of which 25 had not been reported for Cozumel, increasing the known number of rotifers on the island from 17 to 40 species; the species belong to two orders, 11 families and 18 genera. Only two orders of Phylum Rotifera were encountered: Ploimida (11 families) and Flosculariaceae (one family). *Lecane* was the best represented genus, with 12 species, followed by the genus *Brachionus*, with four species. The aquatic system that registered the greatest quantity of species was the Maravillas cenote (19 species), followed by the Uvala and Torre cenote (11 species) and the Sin Barda cenote (nine species). Details of the physical and chemical parameters are shown in Table 2. All the aquatic systems studied are freshwaters: the average conductivity was 409.88 μS/cm^3^ and the average temperature was 26.81°C. The depth was less than 2.27 metres. The dissolved oxygen average was 6.81 mg/l.

New records of species that were found are described below.

**Figure 1.**
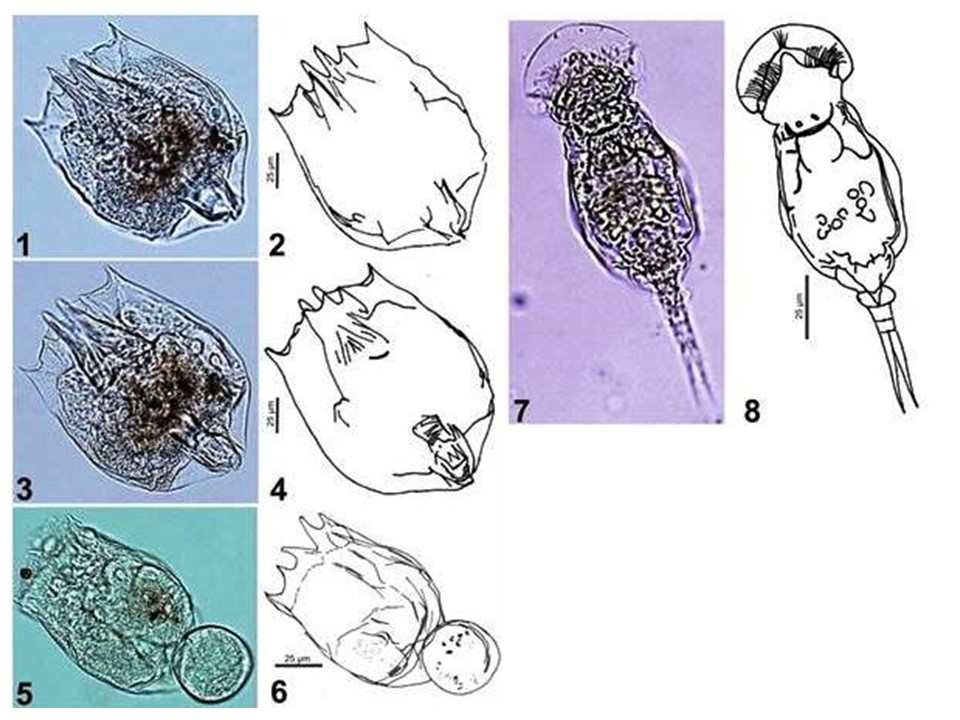
Rotifers of cozumel, 1-4) *Brachionus rubens*; 5, 6) *Brachionus* plicatilis s.l.; 7, and 8) *Squatinella mutica*.

*Brachionus rubens* (Fig. 1). Measured length of 176.55 µm. Anterolateral margin with six spines. Middle spines are longer and sharper than intermediate spines, which are wider at the base. Undulate anterodorsal margin with two striae on each side; elevated central portion with a U-shaped groove. Foot opening with a square aperture; rounded prolongation towards the central body. *B. rubens* is an epizoic rotifer, although it was not observed in association with other species. Even so, *B. rubens* may be associated with insect larvae and cladocera in Cozumel, as observed in other areas.

*Brachionus plicatilis* s.l (Fig. 1). This species belongs to the *B. plicatilis* species complex, wich actually comprises 15 species (Mills et al. 2017) divided into three sizes. The *B. plicatilis* s.l measured 103.21 µm in length, placing it within the smaller-sized *B. rotundiformis* forms category. Spines end in sharp points, protruding from the body in a V shape. García-Morales and Elías-Gutiérrez (2013) reported the DNA sequences of samples of the *B. plicatilis* species complex collected in south-eastern Mexico. However, a detailed analysis of the clonal population structure of the species complex from the Yucatan Peninsula is required.

*Squatinella mutica* (Fig. 1). Ovoid body approximately 110 µm in length; head and ciliary corona located beneath a well-developed semicircular hyaline sheath with an opening of 134 µm. Smooth dorsal and ventral lorica. Posterior portion of body is rounded. Foot formed by two segments with two long toes, each 27 µm in length that end in sharp points without claws or pseudoclaws. Few records exist for this species; in general, its behaviour is not well known.

## Discussion

The number of rotifer species reported in Cozumel has increased to a total of 40, grouped into two orders, 11 families and 18 genera. Segers (1995) mentioned that 380 taxa of the genus *Lecane* have been described worldwide; its preference for littoral aquatic environments and its adaptive capacity enables the wide distribution of this genus compared with other groups. In Mexico, 57 rotifer species have been reported (Cervantes-Martínez et al. 2012) and 46 of these species have been recorded in the south-western region of the country: Quintana Roo, Tabasco, Chiapas and Campeche (Quiroz-Vázquez 2012). Notably, island ecosystems are fragments of natural habitats where species and communities have been separated from the continent and have established, adapted and evolved in a unique manner. For this reason, these environments are critically important for global biodiversity (Lara-Lara et al. 2008).

For example, the rotifer fauna of tropical and Caribbean islands mostly reflect the fauna of the closest continental region (Janetzky et al. 1995). Suárez-Morales and Reid (2003) also supported this idea and suggested that the zooplankton species that inhabit the Yucatan, especially in Quintana Roo, are the result of the geological history of the region. Geologically, Cozumel is considered part of the plate that forms the Yucatan Peninsula (Pacheco and Vega 2008) and shares certain features with this region, such as karstic subsoil and high soil permeability, which facilitate the formation of subterranean caves, cenotes and sinkholes (Lesser et al. 1978). Of the 42 species registered in Cozumel, 35 taxa have previously been reported in the continental region of Quintana Roo, including *Keratella americana*, *Lecane bulla*, *L. crepida*, *L. hastata* and *L. lunaris*, which are considered common in this state. In fact, a large portion of these species are considered cosmopolitan (Elías-Gutiérrez and García-Morales 2011).

The genera *Lecane* and *Brachionus* were the most common taxa represented in this study; this observation agrees with studies performed by García-Morales and Elías-Gutiérrez (2004), Zanatta et al. (2007), García-Morales and Elías-Gutiérrez (2007) in the south-eastern region of Mexico. In fact, these genera are often dominant in the freshwater systems of tropical belts (Keppeler and Hardy 2004). Additionally, environmental factors such as latitude, temperature and conductivity had an effect on species richness of Brachionidae and Lecanidae; for example, latitude had an effect only on species composition of Lecanidae (Sa-ardrit et al. 2017).

The rotifer species identified in this study are largely typical of littoral habitats. This observation may be attributed to the nature of freshwater systems in Cozumel, which are predominantly shallow (< 2 m) and small (18 m in diameter) (Gutiérrez-Aguirre and Cervantes-Martínez 2008), in comparison with the continental aquatic system of the Yucatan Peninsula (47 m in depth and 280 m in diameter) (Cervantes-Martínez et al. 2009). The species diversity of rotifers in Quintana Roo corresponds with the physical and chemical characteristics of its aquatic systems (Elías-Gutiérrez and García-Morales 2011). In general terms, its water bodies are oligotrophic, warm tropical and well oxygenated and have good visibility (Cervantes-Martínez 2005), which differ notably from the meso-eutrophic systems, common throughout the rest of Mexico.

MacArthur and Wilson (1967) mention that, for islands, species richness is directly related to island size and distance from the closest continent. Segers and Dumont (1993) also elaborate this point and affirm that the species richness of islands is related to the distance to the closest continent and territorial extension. For example, Janetzky et al. (1995) registered a total of 177 rotifer species in 60 aquatic systems in Jamaica, an island with an area of approximately 10,991 km^2^ located 630 km from the South American continent. This richness may be attributable to the island's proximity and size.

As previously mentioned, the proximity of Cozumel to the continental coast and its territorial extension could be factors that positively influence its rotifer richness. This idea is in agreement with Yáñez-Mendoza (2008), who studied rotifers in 250 natural pools on the island; approximately 41 pools were major aquatic systems (depths > 3 m). In this study, only 17 sampling sites of lesser depths (> 2 m) were evaluated. As the study of the zooplankton fauna of Cozumel intensifies, the number of monogonot rotifer records for island aquatic environments is likely to increase.

The greatest numbers of species registered in island systems are found in the Neotropical and Eastern biogeographic territories, followed by the Palearctic territory; the lowest number of species is found in the Nearctic territory. Australasia is the most studied island system, in which 687 rotifer species have been recorded. The same study reported 133 rotifer species in the islands of the Pacific Ocean (Segers 2008). Finally, we recommend that additional taxonomic studies be carried out on the zooplankton of Mexican island systems since presumably only a small proportion of the existing taxonomic forms are known (Alonso 1984). Perhaps the potential endemism of island plankton species as a result of the unique characteristics of island environments could also be highlighted (Brown and Lomolino 1998, Lomolino and Weiser 2001).

## Figures and Tables

**Table 1.** List of rotifers from Cozumel: monogonot rotifers had not been reported for Cozumel (*); new records for Quintana Roo (^+^); and new records for Mexico (°). The symbols “X” and “-” indicate presence and absence of the species, respectively. Numbers correspond to the following sampling sites: 1, Pileta UQROO; 2, Maravillas cenote; 3, Sin Barda cenote; 4, Uvala cenote; 5, Palmar cenote; 6, Observation Tower; 7, Caletita cenote; 8, Chenpita; 9, El Colombiano; 10, Chancanaab IV cenote; 11, Ositos cenote; 12, San Gervasio III; 13, Tres Potrillos cenote; 14, Garden cenote; 15, UQROO cenote; 16, Charco cancha UQROO; and 17, Echeverría cenote.

**Taxon**	**Sampling Site**																
	**1**	**2**	**3**	**4**	**5**	**6**	**7**	**8**	**9**	**10**	**11**	**12**	**13**	**14**	**15**	**16**	**17**
**SUBCLASS: MONOGONONTA**																	
**ORDER: PLOIMIDA**																	
**1. FAMILY: Brachionidae**																	
*Anuraeopsis fissa* (Gosse, 1851)*	X	X	X	X	X	-	X	X	X	-	-	X	X	-	-	X	X
*Plationus patulus* (Mϋller, 1786)	-	X	-	-	-	-	-	-	-	-	-	-	-	-	-	-	-
*Brachionus angularis* Gosse, 1851*	X	X	-	X	-	-	-	-	-	-	-	-	-	-	-	-	-
*Brachionus rubens* Ehrenberg, 1838*	-	-	X	-	-	X	-	-	-	-	-	-	-	-	-	-	-
*Brachionus plicatilis* sensu lato*	-	-	-	-	-	X	-	-	X	-	-	-	-	-	-	-	X
*Keratella americana* Carlin, 1943	-	X	-	-	-	X	-	-	-	-	-	X	-	-	-	-	X
*Platyias quadricornis* Ehrenberg, 1832	X	X	-	X	-	-	-	-	-	-	-	-	X	-	X	-	X
**2. FAMILY: Euchlanidae**																	
*Beuchampiella eudactylota* (Gosse, 1886)^+^	-	X	-	-	-	-	-	-	-	-	-	X	-	-	X	-	-
*Euchlanis dilatata* Ehrenberg, 1832*	-	-	-	-	-	-	-	-	-	X	-	-	-	-	-	-	-
**3. FAMILY: Mytilidae**																	
*Mytilina bisulcata* (Lucks, 1912)^+^	-	X	-	-	X	-	-	-	-	-	-	-	-	-	-	-	X
*Mytilina ventralis* (Ehrenberg, 1832)	-	-	-	-	X	-	-	-	-	-	-	-	-	-	-	-	-
*Mytilina mucronata* (Müller 1773)	-	-	-	-	-	-	-	-	X	-	-	-	-	-	-	-	-
**4. FAMILY: Colurellidae**																	
*Colurella adriatica* Ehrenberg, 1831*	-	-	-	-	-	X	X	-	-	-	-	-	-	-	-	-	X
*Lepadella patella* (Mϋller, 1773)	X	X	-	-	X	-	-	-	-	-	-	X	-	-	-	-	X
*Lepadella ovalis* (Müller, 1786)*	-	-	-	X	-	-	-	-	X	-	-	X	-	-	-	-	X
*Lepadella rhomboides* (Gosse, 1886)*	-	-	-	-	X	-	-	-	-	-	-	X	-	-	-	-	-
*Squatinella mutica* (Ehrenberg, 1832)^+^	-	X	-	-	-	-	-	-	X	-	-	-	-	-	-	-	-
**5. FAMILY: Lecanidae**																	
*Lecane aculeata* (Jakubski, 1912)*	-	-	-	X	-	X	-	-	-	-	-	-	-	-	-	-	-
*Lecane bulla* (Gosse, 1851)	X	X	X	X	X	X	X	X	X	X	X	-	-	-	-	X	X
*Lecane closterocerca* (Schmarda, 1859)	-	X	X	-	-	-	-	-	X	-	-	-	-	-	-	X	-
*Lecane crepida* Harring, 1914*	-	-	-	X	-	X	X	-	-	X	-	-	-	-	-	-	-
*Lecane cornuta* (Müller, 1786)*	-	-	-	X	-	-	-	-	X	-	-	-	-	-	-	-	-
*Lecane elsa* Hauer, 1931	X	X	X	X	-	X	-	-	-	X	-	X	-	-	-	X	X
*Lecane grandis* (Murray, 1913)*	-	-	-	-	-	X	-	-	-	-	-	-	-	-	-	-	-
*Lecane hamata* (Stokes, 1896)	-	X	X	-	-	-	-	X	-	X	X	-	-	-	-	-	-
*Lecane obtusa* (Murray, 1913)*	-	-	-	-	-	-	-	-	-	X	X	-	-	-	-	-	-
*Lecane pyriformis* (Daday, 1905)*	-	-	-	-	-		-	-	-	X	-	-	-	-	-	-	-
*Lecane quadridentata* (Ehrenberg, 1830)	X	X	X	X	X	X	-	-	-	-	-	X	-	-	X	-	X
**6. FAMILY: Notommatidae**																	
*Cephalodella forficula* (Ehrenberg, 1838)*	-	X	-	-	-	-	-	-	X	-	-	-	-	-	-	-	-
*Scaridium botsjani* (Dames & Dumont, 1974)*	-	-	-	-	-	-	-	-	-	X	-	-	-	-	-	-	-
**7. FAMILY: Trichocercidae**																	
*Trichocerca pusilla* (Jennings, 1903) *	X	X	X	-	-	-	-	-	-	-	-	-	-	-	-	-	-
**8. FAMILY: Synchaetidae**																	
*Polyarthra cf. dolichoptera* Idelson, 1925*	X	X	-	-	-	-	-	-	-	-	-	-	-	-	-	-	-
*Polyarthra vulgaris* Carlin, 1943*	X	X	X	-	-	-	-	-	X	-	-	-	-	-	-	-	X
**9. FAMILY: Asplanchnidae**																	
*Asplanchnopus multiceps* (Schrank, 1793)	-	-	-	-	-	-	-	-	-	-	-	-	X	-	-	-	X
**10. FAMILY: *Dicranophoridae***																	
*Dicranophorus epicharis* Harring & Myers, 1928^+^	-	X	-	-	-	-	-	-	X	-	-	-	-	-	-	-	-
**ORDER: FLOSCULARIACEAE**																	
**11. FAMILY: Testudinellidae**																	
*Testudinella patina* (Hermann, 1783)*	-	-	X	X	-	X	-	-	-	-	-	-	-	-	-	-	-

**Table 2.** Geographical location and physical and chemical characteristics of the study sites. ND = no data.

**No**	**Name of Site**	**Latitude N**	**Longitude W**	**Type of Sample**	**Conductivity (μS/cm^3^)**	**Dissolved Oxygen (mg/l)**	**Max. depth (m)**	**Temperature (°C)**
1	Pileta UQROO	20°29'17.9"	86°56'23.2"	Limnetic	539.5	7.27	1.1	27.15
2	Cenote Maravillas	20°29'28.1"	86°56'50.2"	Limnetic	230.35	14.31	1.2	21.35
3	Cenote Sin Barda	20°29'11.2"	86°57'19.9"	Limnetic	500.1	1.91	2.1	28.4
4	Cenote Uvala	20°22'8.0"	86°58'7.6"	Littoral	300.3	1.78	< 1	25.3
5	Cenote Palmar	20°21'59.8"	86°58'19.2"	Littoral	350.1	1.8	< 1	26.4
6	Torre de Observación	20° 32'31.2"	86°52'47.5"	Limnetic	767.9	12.45	2.1	25.8
7	Cenote Caletita	20°29'42.1"	86°57'47.6"	Littoral	567.78	6.89	2.5	24.7
8	Cenote Chenpita	20°22'59.8"	86°58'5.0"	Limnetic	458.6	19.5	5	29.1
9	Cenote "El colombiano"	20°21'56.17"	87°58'45.3"	Littoral	210.5	5.6	2.3	30.5
10	Chankanaab IV	20°26'31.67"	86°59'40.14"	Littoral	367.7	6.27	2.16	27.81
11	Charco Ositos	20°29'28.62"	86°56'24.55"	Littoral	N/D	N/D	N/D	N/D
12	San Gervasio II	20°26'32.49"	86°51'43.51"	Littoral	595.2	4.51	2	27.6
13	Tres Potrillos	20°27'0.5"	86°59'15"	Limnetic	N/D	N/D	2.25	25.7
14	Cenote Gardner	20°29'42.2"	86°57'06.2"	Limnetic	N/D	2.01	<1	N/D
15	Cenote UQROO	20°29'19.22"	86°56'26.36"	Limnetic	230.35	6.87	< 1	27.15
16	Cancha UQROO	20°29'15.80"	86°56'30.33"	Littoral	N/D	1.45	< 1	28.5
17	Cenote Echeverria	20°29'14.95"	86°57'11.28"	Littoral	210.1	9.63	< 1	N/D

## Hosting institution

Centro de Investigación Científica de Yucatán A.C
